# “This is a kind of betrayal”: a qualitative study of disability after breast cancer

**DOI:** 10.3747/co.v16i3.389

**Published:** 2009-05

**Authors:** R. Thomas–MacLean, A. Towers, E. Quinlan, T.F. Hack, W. Kwan, B. Miedema, A. Tilley, P. Graham

**Keywords:** Breast cancer, psychosocial effects, qualitative study, disability, arm morbidity

## Abstract

**Objective:**

We proposed to document the effect of arm morbidity and disability in 40 Canadian women who were 12–24 months post breast cancer surgery.

**Methods:**

We completed 40 qualitative interviews as one component of a multidisciplinary national longitudinal study of arm morbidity after breast cancer (*n* = 745) involving four research sites (Fredericton/Saint John, Montreal, Winnipeg, Surrey). During semi-structured interviews, participants who had reported arm morbidity and disability in earlier surveys were asked to discuss the effects of these conditions on everyday life.

**Results:**

The interviewees reported making major adjustments to paid and unpaid work, which often involved the assistance of family members, thus demonstrating the effect of disability. Interview data resulted in the creation of a model that addresses arm morbidity and disability, and that holds implications for health care professionals.

**Conclusions:**

Based on the interview findings, we conclude that a robust measure of disability after breast cancer should be developed. In the absence of a validated measure of the effect of disability, evaluating qualitative responses to questions about everyday activities could provide the impetus for provision of physical therapy and emotional support.

## 1. INTRODUCTION

Much research has been devoted to the topic of breast cancer, and yet issues surrounding arm morbidity and disability remain sparsely documented, with the possible exception of lymphedema. Although women who experience lymphedema indicate that the condition has a major effect on everyday life^1^, little is known about pain and range of motion (rom) restrictions. Nonetheless, the potential effects of arm morbidity on everyday life for women is immense. For instance, one of our participants identified arm problems as a “kind of betrayal,” comparable to the emotions she felt when first diagnosed with breast cancer.

Preliminary findings from our study predict that between 30% and 50% of women with breast cancer will experience some form of arm morbidity. With 22,000 Canadian women being newly diagnosed with breast cancer each year, and overall survival rates cited at 86% (higher for stage i cancer), arm morbidity and disability stand to affect vast numbers of women. Qualitative research can illuminate the social impacts of such conditions, confirming the need for a transdisciplinary approach to research, treatment, and policy. The present article reports on the qualitative findings of a longitudinal study examining three types of arm morbidity—pain, rom restrictions, and lymphedema—in a subsample of 745 Canadian women.

## 2. BACKGROUND

Broadly, recent research on breast cancer survivor-ship has started to acknowledge the long-term social effects of the illness. A synthesis of the literature illustrates that women with breast cancer, as compared with those without, experience a decline in physical function^2^. Recent research also shows that an economic impact is associated with breast cancer^3^. Others authors have noted that long-term disabilities may be associated with the experience of cancer; returning to work is often complex for cancer survivors^4^–^6^. Yet, within the biomedical literature, little consensus can be found concerning the appropriate measures for diagnosing lymphedema, let alone the best methods for managing this condition and its psychosocial effects^7^–^11^.

The advent of sentinel lymph node biopsy (slnb) has led some clinicians to assume that lymphedema is no longer an issue for breast cancer patients because, according to clinical trials, slnb reduces the rate of lymphedema as compared with axillary lymph node dissection (alnd). Nevertheless, alnd must still be completed for patients with a positive slnb, and a risk of lymphedema remains in those who undergo slnb alone^12^. Additionally, the surgical uptake of slnb may vary by geographic location because of the varying rate of adoption of new surgical techniques. For instance, within the United States alone, breast-conserving surgery is influenced by factors such as region, type of hospital, and the surgeon’s years of practice^13^. How widely slnb has been adopted in Canada is not known, but within the first half (approximately 46%) of our sample (n = 745) enrolled in a mixed-methods study of arm morbidity, about 77% had undergone an alnd or a combination of slnb and alnd^11^,^a^. Finally, because slnb is a relatively new surgical technique, and because survival rates for breast cancer have increased to approximately 86% since the 1970s, it is reasonable to assume that a large population of breast cancer survivors will have experienced an alnd at some point. Additionally, other types of arm morbidity such as pain and rom restrictions have not been fully addressed. A common assumption appears to be that, despite evidence to the contrary^7^,^8^,^10^,^14^, such symptoms do not affect women’s lives after breast cancer surgery.

In Canada, some cancer rehabilitation programs have emerged^15^, but no national rehabilitation program for cancer of any type exists, and despite a national health care system, cancer follow-up care is inconsistent^16^, often varying by location. Moreover, cancer follow-up care tends to focus on the primary illness rather than on the effects of that illness or on the treatment of subsequent disabilities. Possible interventions for lymphedema are still exploratory^17^, and interventions for pain and rom restrictions are under-researched.

Highlighting the effects of disabilities on the social aspects of the lives of breast cancer survivors remains an important task. Researchers have suggested that mixed-methods studies are well suited to establishing clinical significance in relation to quality of life after cancer^18^. Qualitative findings from our study demonstrate the manner in which arm morbidity intersects with complex social relations, particularly in areas such as a woman’s paid and unpaid work and in familial relationships.

## 3. PATIENTS AND METHODS

### 3.1 Study Setting

Our study was conducted at four sites in Canada: Fredericton/Saint John, New Brunswick; Montreal, Quebec; Winnipeg, Manitoba; and Surrey, British Columbia. Participants from these sites represent both urban and rural areas.

### 3.2 Methods

A qualitative approach (modified grounded theory) was used to draw upon the details of everyday experiences. Interview data was then analyzed using NVivo 7 (QSR International, Doncaster, Victoria, Australia), a qualitative data analysis program, to generate a model.

### 3.3 Research Ethics Board Certification

Our research was approved by the appropriate research ethics board at each study site. Participants signed informed consent forms before data collection commenced.

### 3.4 Inclusion Criteria

For the study overall, these were the inclusion criteria:

- Female sex
- Age of 18 years or older
- English- or French-speaking
- Able to provide informed consent
- With unilateral breast cancer
- Diagnosed with stage i–iii disease

For the qualitative interviews, 40 women with arm morbidity (that is, pain, rom restrictions, or lymphedema or self-reported swelling) were invited to participate.

Pain was measured using the McGill Pain Questionnaire, with the Present Pain Index being the key indicator of pain for the purpose of interviewee selection.

Restrictions in rom were assessed by research associates who used a goniometer to measure abduction and rotation. Those measurements were then compared with established cut-offs for rom impairment: that is, less than 80 degrees for rotation and less than 170 degrees for abduction.

Lymphedema was assessed using sequential circumferential arm measurements. The measurements were then entered into a spreadsheet that used a truncated cone formula to calculate the percentage volume increase in the affected arm, with 5% being indicative of lymphedema.

Additional details about the quantitative aspects of, and clinical assessments associated with, this study can be found in Thomas–MacLean *et al.*^11^

### 3.5 Recruitment Process

Patients were consecutively recruited from oncology and surgical clinics by onsite collaborators. Data collection was conducted every 6 months, alternating between clinical assessments and telephone interviews. Medical chart data were also collected to provide information about stage of breast cancer, treatment, and other disease-related statistics.

We interviewed 10 women at each of the four research sites, which represent both urban and rural areas in the eastern, central, and western parts of Canada. Interviews were conducted approximately 12–24 months post surgery. The selection of potential interview candidates was informed by the reported experiences of arm morbidity symptoms at the first data collection point (6–12 months post surgery) and by the demographic data (to ensure that diverse perspectives were being elicited). Guided by this theoretic approach to sampling, the research associates approached women with arm morbidity—that is, pain, rom restrictions, and self-reported swelling or clinically observed lymphedema (or both)—at their sites and invited those women to participate in the interviews. Tables i–iii summarize the composition of the sample of 40 interviewees (that is, demographics, treatment variables, and reported types of arm morbidity).

### 3.6 The Interview

Interviews were digitally recorded. The interview guide consisted of open-ended questions about a variety of topics, beginning with a request for descriptions of arm morbidity symptoms. Research associates then asked the participants about possible effects in a variety of domains, including work, family, and leisure activities.

### 3.7 Data Analysis

All interviews were transcribed verbatim. Data analysis was completed by three research team members who first read all the transcripts holistically, with a view to learning about the effects of arm morbidity on various aspects of the women’s lives, including paid or unpaid work, family, and leisure activities. Then, following established, grounded theory guidelines, transcripts were read line-by-line to extract significant statements from the interviews^19^. Subsequently, the three team members engaged in several discussions about emerging themes; these discussions resulted in a coding framework. Significant statements were used to generate specific codes, which were then used to create a schema within the qualitative data analysis program (NVivo 7). This process resulted in a model. The key themes—that is, those described in the most detail or described by participants as having the greatest impact—were paid work, unpaid work, and leisure activities.

## 4. RESULTS

### 4.1 Profile of the Interviewees

Table i outlines the participant demographics, which includes age categories. The average age of the interviewees was 52 years. Almost half the participants (47.5%) reported incomes over $80,000, and 72.5% had completed some postsecondary education. Many of the interviewees (40%) had children living at home. Almost one third (30%) lived in rural areas.

Table ii provides treatment-related data. Of these interviewees, 77.5% had either alnd or both alnd and slnb. Most had been treated with radiation and chemotherapy (70% and 75% respectively).

Table iii shows that, with respect to arm morbidity, some of the interviewees (22.5%) had clinically measured swelling congruent with definitions of lymphedema.

Many of the interviewees (72.5%) had self-reported swelling: that is, they responded positively to a question asking whether they had experienced swelling of the arm, shoulder, or hand in the last 6 months. Experience of pain was reported by 62.5% of the interviewees, and 82.5% reported experiencing rom restrictions. Most of the interviewees (78%) reported more than one type of arm morbidity (that is, clinically measured lymphedema, rom restrictions, pain, or self-reported swelling).

Most of the interviewees (80%) had discussed treatment for arm morbidity with a health care professional, but many (47.5%) had not received treatment (Table iv). Many of the interviewees (75%) also reported being involved in exercise with the arms three or more times weekly, which may help to minimize arm morbidity symptoms.

### 4.2. Key Themes Related to Disability

#### 4.2.1 Theme 1: Paid Work

In discussions about the effects of arm problems on paid work, women in a variety of occupations (scientist or artist, for instance) reported a need to modify or cease work. Participants also spoke of the challenges associated with reduction in income and the roles of co-workers.

With reference to changing or ceasing work, one woman, an artist, said, “I used to paint, but now this situation does not help me, so I don’t really work.... I cannot paint ... so I cannot work” (M093)^b^.

Ceasing paid work was not uncommon. One woman said that she was on disability and could no longer do her job as a result of arm problems (W002). When asked about the effects of arm problems on paid work, another participant responded, “I have stopped work for a week because ... the doctor told me to rest and keep my arm raised higher than my heart” (M105). Another participant mentioned that she experienced swelling and pain after writing or working at the computer, and that her job involved a great deal of both (M243). This same participant said she had worked at four jobs simultaneously before her breast cancer and subsequent arm problems. She complained that her life now was “boring.”

Other participants wondered if they would be able to continue paid work. One participant anticipated problems, noting that her hand had “started to give [her] a little trouble after doing a lot of computer work,” but that she had not yet reached a point where she was unable to do her job (W041). Likewise, this participant spoke of the possibility of having to retire from her job much earlier than she had expected: “Retirement is very final.... I never thought I’d see myself at age 49, having to say I can’t work anymore. Because I love work. If I didn’t like work, it would be an easy decision to make” (W037).

Other participants found that they were still able to do the work that they had done in the past, but only with the support of co-workers. For example, one woman said that her co-workers did any lifting that would usually be required of her (W070).

Thus, with reference to paid work, women reported ceasing or modifying work, anticipating a cessation of paid work, and facing problems associated with a loss of income and with the role of co-workers.

#### 4.2.2 Theme 2: Unpaid Work

Completing unpaid work was also described as problematic for some participants.

Many aspects of unpaid work were described as challenging or impossible for the interviewees. One woman said, “I cannot lift, except with my left arm. I cannot do big jobs. With cooking, when I hold the skillet sometimes, it falls from my hand” (M105). This participant went on to say that she had also experienced difficulties with vacuuming, washing windows, sweeping, and even holding a book. At the time of the interview, she was relying on a paid assistant to complete these tasks. Other participants also spoke of difficulties with doing laundry, shovelling snow, or completing other tasks that involve heavy lifting.

Some women relied on their partners in ways similar to the reliance on co-workers in paid positions. A participant said of her partner, “I’m very fortunate, because my husband will do things that I can’t do ... window washing, washing floors, and walls” (W033).

Along with their partners, the women in our study also relied on friends for help with unpaid work. One participant said, “If I go shopping with my girlfriend, she carries the parcels” (F011).

Other participants compensated for their inability to complete domestic work. One woman said, “We actually bought a different kind of vacuum, because I found the other one was hard on me” (F016). Another woman said that, to reduce the amount of housework she was doing, she and her partner had purchased a dishwasher (W002).

Therefore, as with paid work, participants reported that they were unable to complete tasks they had previously been able to do, and they relied on other people to assist them with unpaid work. The descriptions of assistance provided for unpaid work points to the intersection between unpaid work and relationships with family, which were also discussed by participants.

#### 4.2.3 Theme 3: Family Relationships

Participants spoke of a number of relationships that were affected by disability after breast cancer, including those with partners, children, and grandchildren.

Some women noted that their partners were very supportive and helpful. One called her partner a “sweetheart” and mentioned that her partner carried her purse for her (W073). This woman also noted that her partner built a special padded platform for use in the car, so that she could elevate her arm while travelling. Another participant said that her partner helped with all heavy lifting and cited “draining spaghetti” as an example (W002).

Although some women’s partners were supportive, the partners also experienced their own limitations because of illness. For instance, one woman noted that her partner helped with more household tasks, but that he had his own limitations because of emphysema (W037).

Relationships with partners were not the only ones being affected by arm morbidity. One participant said, “I cannot pick up my granddaughter” (M093). Another woman also spoke of the physical nature of relationships with grandchildren and of her inability to fully participate in that aspect of their lives: “I have seven grandchildren and they are very physically active and physically affectionate grandchildren. They want to be hugged and picked up and kissed and all that stuff, and I do find some difficulty in lifting them” (S114).

The intersection between unpaid work and family also emerged in some descriptions. One participant said that she had had to reflect more about the ways in which she completed tasks *and* interacted with others: “Well, physically I have to be more conscious about what I do. I can’t just unconsciously go through life, where you normally would just pick up a bag, or pick up a kid. I find I have to stop and think. There is an element of stress in that, just having to be careful or having to wait for somebody to do something for you” (S118).

In summary, women reported a number of changes, both positive and negative, to various relationships with their partners, children, and grandchildren.

## 5. DISCUSSION AND CONCLUSIONS

**FIGURE 1:**
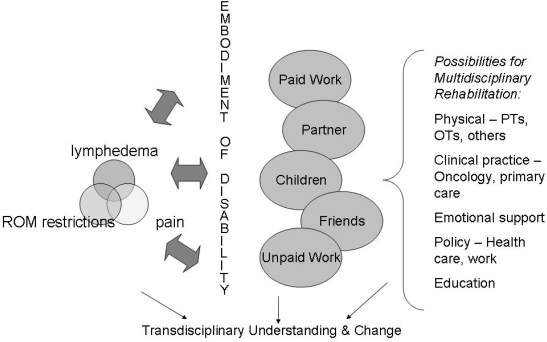
*Model of disability after treatment for breast cancer and of implications for practice.* *rom = range of motion;* *pts = physical therapists;* *ots* *= occupational therapists.*

Our findings speak to the complexity of the social effects of arm morbidity after breast cancer. The processes associated with disability after breast cancer are illuminated through discussions of paid work, relationships with family, and unpaid work. All demonstrate the need for rehabilitation. The model (Figure 1) resulting from our qualitative findings illustrates some of the intersections of the various domains of women’s lives and the combined social and physical effects of disability.

Paid work became challenging for many participants and disrupted identity. Without rehabilitation programs in place, it is quite possible that arm problems and disability will worsen over time. Our findings show that the loss of paid work may be quite upsetting, particularly for women who previously relied on their income from paid work and for women who enjoyed working outside of the home. It should be noted that the income level of these particular interviewees was relatively high, which may have had an effect on the ability of the participants to compensate for limitations in paid work. However, this income level is not reflective of the larger sample from which the interviewees were drawn, and productivity is discussed further in another manuscript examining quantitative findings^6^.

Participants also faced challenges with unpaid work and missed being able to complete it. Work, whether paid or unpaid, was meaningful to many participants and central to their identity. The bidirectional relationship between work and the embodiment of disability (which encompasses physical, social, and psychological aspects) is illustrated within the model.

The reliance by women on their partners may also prove problematic, even though many women described their partners as being supportive. Partners may also have chronic illnesses or disabilities, some of which may emerge over time. So, although some partners were able to assist the women in our study, such assistance may later prove to be more difficult if arm morbidity symptoms worsen or as partners become ill themselves. A woman who has a supportive partner may face challenges if the relationship ends, or if the partner dies. It is not uncommon for women to outlive male partners, in which case, the effects of disability may become more profound. Similarly, women without partners may also face more challenges with paid and unpaid work, as would women without supportive employers. In addition, women with low incomes would not have recourse to some of the solutions that certain participants adopted for assistance with unpaid work—for example, buying a dishwasher or a new vacuum. In short, as demonstrated by our model, disability after breast cancer has many, varied, and interconnected social effects related to paid work, unpaid work, and family as well as to physical symptoms and emotional effects.

Our work also points to the challenges posed in developing measures of disability. We used a measure of disability in the quantitative component of our study, but the dash (Disability of Arm–Shoulder–Hand) outcome measure does not allow for the possibility of minimizing the effects of disability through the assistance and support of family and friends. Also, the dash measure does not address any changes or adaptations to work that people might make, nor does it gauge supportive features of the work environment (for example, a unionized workplace or an understanding employer). Nonetheless, our findings make it clear that health professionals could, in the absence of a robust measure of disability, begin to assess the effects of disability on the lives of their patients simply by asking questions about paid and unpaid work and relationships with family.

Addressing arm morbidity and disability after breast cancer will require complex and multidisciplinary approaches, as indicated in our model. The development of a suitable measure for the effects of disability that includes some recognition of social context would also help to transcend traditional disciplinary boundaries that do not always recognize the interrelated features of disability. In terms of health practices, establishing social effect represents one step toward a rationale for the establishment of rehabilitation programs.

## Figures and Tables

**TABLE I:** Demographics of the study participants

*Characteristic*	*(*n*)*	*(%)*
Age		
30–39 years	4	10
40–49 years	14	35
50–59 years	14	35
60–69 years	6	15
70–79 years	2	5
Family income		
>$20,000	6	15
$20,001–$40,000	1	2.5
$40,001–$80,000	6	15
$80,000+	19	47.5
Not reported	8	20
Education		
Junior high school	2	5
High school	9	22.5
College	13	32.5
University	16	40
Children at home		
0	24	60
1	3	7.5
2	11	27.5
3	2	5
Location		
Rural	12	30
Urban	28	70
Occupational category		
Arts and culture	3	7.5
Business and finance	5	12.5
Education	5	12.5
Health	5	12.5
Management	5	12.5
Sales	1	2.5
Science	1	2.5
Social science	1	2.5
Trades	2	5
Not in workforce	12	30

**TABLE II:** Treatment received by the study participants

*Treatment*	*(*n*)*	*(%)*
Surgery		
Radical mastectomy	1	2.5
Modified radical mastectomy	12	30
Partial mastectomy	27	67.5
Lymph node dissection		
Sentinel node	8	20
Axillary only	23	57.5
Sentinel node and axillary	8	20
Unknown	1	2.5
Radiation		
No	2	5
Yes	38	95
Chemotherapy		
No	10	25
Yes	30	75

**TABLE III:** Arm morbidity among the study participants

*Symptom*	*(*n*)*	*(%)*
Lymphedema		
No	31	77.5
Mild	9	22.5
Restricted range of motion		
No	7	17.5
Yes	33	82.5
Pain		
No	15	37.5
Mild	16	40
Discomforting	9	22.5
Self-reported swelling		
No	11	27.5
Yes	29	72.5

**TABLE IV:** Arm morbidity treatment

*Treatment*	*(*n*)*	*(%)*
Discussed treatment		
No	8	20
Yes	32	80
Received treatment		
No	19	47.5
Yes	21	52.5
Days per week participated in exercise involving arms		
Fewer than three	10	25
Three or more	30	75

## Footnotes

a Hack TF, Kwan W, Thomas–MacLean R, *et al.* Predictors of arm morbidity following breast cancer surgery. In preparation.

b Participants are identified by site (M, Montreal; F, Fredericton/Saint John; S, Surrey; W, Winnipeg) and a participant identification number.
